# Clinical Reports from Prague and Berlin

**Published:** 1878-12

**Authors:** W. S. Caldwell

**Affiliations:** Paris


### Foreign Correspondence.

### CLINICAL NOTES FROM PRAGUE AND BERLIN.

It is strange that the farther one gets from the source of the Lister method of antiseptic surgery, the more firmly the profession believe in its efficacy. Mr. Spence, Mr. Lister's former colleague in Edinburgh, is one of the bitterest opponents of the system that I have ever heard upon the subject. In the performance of an amputation at the hip joint, I was surprised to see him using the spray, after what I had heard him say in opposition to, and almost in ridicule of the whole system.

His explanation was that he did so in deference to a public sentiment in its favor, as the operation was one of the most dangerous known to surgery.

The patient recovered and the advocates of the carbolized spray asserted that it was the only case of the kind that Mr. Spence had operated upon successfully for many years. In London, fifteen months ago, most of the surgeons did not use the spray in their operations. In Vienna it is generally used, though often very imperfectly. In Prague the system is carried out in all its details. They even sent an assistant to London, the past winter, to learn from Mr. Lister himself all the minutiæ of the process.

While in Prague, I spent most of my time in the surgical and gynæcological wards.

### Cæsarean Section.

Prof. Breisky, of this city, has lately done this operation by the new method of removing the entire uterus, after the manner which I described in a former letter as done by Carl Braun, of Vienna. Prof. Breisky tells me that this is the seventeenth

Cæsarean section that has been done here in Prague during the last 35 years, and that this is the only patient who has recovered. The operation was done strictly antiseptically.

I saw the stump dressed frequently, and the most scrupulous care was used to prevent its being exposed for a moment to the air without the application of the spray.

Prof. Breisky remarked that the procedure was rather expensive for a pauper patient, as the dressings had cost $20 for the first two weeks.

I asked the doctor what he thought of Prof. Thoma's new method of doing this operation. His reply was that with the favorable results obtained by this mode of operating, he thought that any other plan was absolutely criminal.

### Emmet's Operation, as it is called here, of paring the edges of the cervix uteri where it has been torn during labor, and bringing the parts together by means of silver wire sutures, Prof. Breisky has done thirty-two times during the last eighteen months. He tells me that the benefits that are supposed to be derived from the operation by its advocates are, in his opinion, greatly over-estimated. He finds, from an extensive observation of cases, that many women who have laceration of the muscular fibers of the cervix uteri have none of the morbid symptoms which this operation is supposed to remedy: and that, furthermore, few of the cases upon which he operated were benefited further than could be accounted for by their necessary confinement in bed, and the inflammatory reaction that must follow after so considerable an operation.

### Parametritis.

In the treatment of this affection, Prof. Breisky's idea is to begin early, and vigorously combat the tendency to the formation of pus. To fulfill this indication, he uses the tincture of iodine, applying it not only to the pelvic walls outside, but, putting the vaginal walls well upon the stretch with a bivalvular speculum, he applies the remedy high up on the vaginal mucous surface, as strong as can be borne by the patient. He makes the applications twice in the twenty-four hours.

### Erysipelas.

In the surgical wards in Prague, a paste composed of equal parts of tar and alcohol is the only remedy used locally in the treatment of this disease. It is spread upon a cloth large enough to cover the surface affected, and to extend a short distance on to the healthy skin. The applications are only changed when they become dry, as it is considered desirable not to expose the parts to the air any oftener than is absolutely necessary. They give at the same time internally one gram of quinia in the twenty-four hours, divided into four doses.

The tincture of the chloride of iron, which is so generally given for this disease in America, and which Prof. Gross taught me, fifteen years ago, was an absolute specific if given in sufficiently heroic doses, I have not found used at all in the wards of any of the hospitals that I have visited in Europe.

Dr. Goldthammer, of the Bethanien hospital at Berlin, applies locally a three per cent. carbolized olive oil. He covers the parts thickly with this mixture, and then envelops them with a heavy layer of sheet wadding, and does not change the dressing oftener than once in the twenty-four hours. He gives nothing internally but wine and a generous diet, being careful at the same time that the patient has plenty of fresh air.

In Prof. Frerich's wards in the Charité, in Berlin, I saw a number of cases of erysipelas and observed closely his manner of treating them. Among them, was a robust girl aged 22 years, who had the disease in a very severe form. Prof. Frerich made her case the subject of a clinical lecture.

"This," he said, "gentlemen, is a case of diffused erysipelas, and is one of the most troublesome and unsatisfactory diseases that we have to treat. She has now been in the hospital eight days. The eruption, which began on her face, has gradually run down until it has now reached the middle of her thighs, and when it has run out at the ends of her toes, it may light up again where it first began, and traverse the whole body a second and even a third time.

"During two days of this time her temperature fell to the normal standard, but this morning it is 40.20. It is one of the diseases in which the temperature curves are governed by no known laws. To-morrow it may be normal, or it may even exceed that which we have to-day.

"It would consume an entire hour, if I should attempt to give you even a catalogue of the different remedies, local and constitional, that have been brought forward and highly extolled in the treatment of this disease, during the forty years of my professional life; but as they have each proved absolutely worthless in my hands, I will not burden you with their enumeration, further than to remark that all *irritating substances*, such as the tincture of iodine, the nitrate of silver, and the like, applied with a view to limiting the spread of the disease, are worse than *useless*, they are absolutely *hurtful*, for while they entirely fail to fulfill the indication for which they are applied, they add to an already existing inflammatory condition of the skin.

"The last plan of treatment that has been brought to our notice was claimed to be founded on something more than simple empiricism. The disease, as was asserted, being produced by the impregnation of the cuticle with certain bacteria, we had only to resort to that great destroyer of the lower forms of animal life, carbolic acid, to effectually arrest its progress.

"We were told that the hypodermic injection of this remedy upon the borders of and just beyond the confines of the eruption would completely head off the disease in its onward march. In my wards here in the Charité we have tried this plan of treatment most effectually, and I can assure you that we have not only found it of no use, but absolutely, and often to a high degree, *injurious*. It has no effect at all in arresting the spread of the disease; besides, we have had cases where abscesses have followed its use that were troublesome to manage, and highly prejudicial to our patients.

"If we are to discard all these new and old plans of treatment, then what are we to do? Unburden your minds of all specific remedies, and treat your case symptomatically entirely. If this patient cannot sleep, we will give her opium or chloral, whichever seems best fitted to fulfill the indication in her particular case. When her temperature is down, and she appears exhausted from the previously existing hyper-pyrexia, we will give her wine, camphor and the like.

" As her temperature is very high at present, we shall prescribe for her to-day 1 gram of quinia to be divided into three doses, one to be given every eight hours. Besides this we are using cold baths of a temperature of from 18° to 24°, repeated from two to six times a day, according to the temperature that we have to combat.

" We are keeping the parts that are the seat of the most active stage of the disease constantly enveloped in cold compresses, from water of a temperature of from 10° to 16°, and this is all we are doing, or shall do."

I watched this patient every day, after the above lecture was given, while she remained in the ward. The disease did not entirely disappear until the fifteenth day after she entered the hospital. On the 10th day her temperature was normal for twelve hours, and on the 12th day she had no fever for the same length of time, but after each intermission she had an exacerbation in which her temperature rose to above 40°.

### Volvulus.

Mrs. E., a married woman, aged 43 years, suffering from complete obstruction of the bowels, was brought into the lecture-room and her case made the subject of some clinical remarks by Prof. Frerichs. She had had no evacuation from her bowels for twelve days, was bathed in a cold perspiration, her countenance expressed extreme suffering, her whole aspect seeming to portend a speedy dissolution. She vomited everything she took into her stomach, and the matters ejected were frequently mixed with stercoraceous ingredients. Prof. Frerichs said :

" Gentlemen, the first question that presents itself in connection with this case, is the location of the obstruction ; and the second point for consideration is its character. As we look at her abdomen and observe the irregular peristaltic contractions that take place, we see that they are mainly confined to the central portion of the bowels, near and around the umbilicus.

" As we follow around the course of the colon, we find this portion of the intestinal track undistended and comparatively free from any unnatural movement. This leads us to infer that the obstruction is in the small intestines, probably in the jejunum. Now as to its nature. Upon this point we shall have to speak with some reserve.

"What do her symptoms lead us to infer? We have not been able to find any circumscribed hard mass or tumor, even by the most careful external exploration. We therefore exclude both hardened fæces and gall stones as the cause of obstruction in this case. Our next most frequent cause of an acute attack like this, is either intussusception or a twisting of the gut upon itself. We believe the cause of the difficulty in this case, to be due to one of the last two conditions, but to which of these conditions we are unable to say.

"Of course we would not think of giving a patient like this a cathartic. If we were *certain* that we had intussussception, we would give an opiate, to quiet all peristaltic contractions of the intestines; but as this remedy would be contraindicated if we have simply a twisting of the gut upon itself, we shall discard this remedy also.

"We shall use for this patient ice and ice water, and these alone. By means of a Hegar irrigator we shall throw as much ice water into the bowels, per rectum, as we can possibly do without fear of their over distention. We have used in this case two litres of water at each irrigation, and have repeated this every 4 to 6 hours. We give her by the mouth all the ice that she can eat, and nothing else."

From the general aspect of this patient I had scarcely a doubt but that she would soon die, and so I watched the autopsies at Prof. Virchow's pathological rooms for two days, expecting to be able to see the diagnosis in her case cleared up by a post-mortem, but as she did not make her appearance, I went in search of her, to the ward in which was her bed, on the third day, and found to my surprise that she was sitting up in bed, eating a plate of soup, and was convalescing nicely.

### Fractures of the Femur.

After observing the animated discussions that have lately occurred on the other side of the Atlantic, between Prof. Sayre on the one hand and Prof. Frank Hamilton on the other, as to the possiblity of obtaining perfect results in the treatment of fractures of the femur, I have been lead to carefully watch the management of this accident in the hospitals in Europe, not only as to the mechanical appliances used, but also as to the results said to be obtained. Although the limits of this article will not allow me to go extensively into a review of this very important question, yet I will give the followig brief summary of what I have observed.

The first point that strikes me as a deviation from what is so stringently taught by most of our surgeons, is that there are *generally* no active measures used to produce extension and counter-extension in the management of these cases. Buck's extension apparatus (or the American method, as it is called here) is used very infrequently indeed. The surgeons in St. George's Hospital in London are the only ones whom I have found who use it *generally*, and they only do so when the fracture is very oblique and the shortening very considerable. When I have asked surgeons why they did not adopt this simple means in the treatment of this accident, they have generally found fault with it on two accounts; first, its inconvenience to the patient by producing pain, etc., and the liability of non-union following its use if the weight used was very heavy. The appliances that are used in the treatment of this accident are almost as numerous as the men who treat it; yet they very generally fail in the one thing that is considered needful with us, and that is, in producing any considerable extension and counter-extension.

At the Bethanien Hospital, in Berlin, I saw a number of cases of this kind being treated, and I got some items that would delight Prof. Hamilton, and furnish him with some valuable paragraphs for the next edition of his work on fractures and dislocations.

Prof. Wilms says that he tried effectually the application of the plaster of Paris dressing with a view of keeping up counter-extension in the perineum; and that whenever he effected this object, it was at the expense of great suffering and inconvenience to his patients, and that in several instances it produced extensive sloughing in this region. He, however, uses the plaster of Paris dressing, not extending it *internally* sufficiently high to press upon the perineum, but carrying it up on the outward aspect of the limb as high as the crest of the ilium, extending the bandage around the entire pelvis, but not applying the *plaster* except to the outward aspect of the dressing. He further says that extension and counter-extension are *generally* not necessary in the treatment of these fractures; that the main point is to *adjust* your fracture *well*, and then keep the parts quiet. To effect this object, he anæsthetises his patients with chloroform before he attempts to adjust the parts. This, he claims, takes off all muscular spasm, and enables him to do his work well. He applies his bandage under the influence of the anæsthetic, an assistant keeping up extension until the bandage dries sufficiently to be firmly set. To facilitate the rapid drying of the dressing, he mixes his plaster of Paris in hot water.

Prof. Wilms says that his results are good, and when I asked him how good, he said that the shortening was generally under one inch.

In the cases I saw treated in Prof. Langenbeck's clinic, the plaster dressing was only applied as high as the hip joint, the perineum not being pressed upon. A long, external, wooden, straight splint extended from above the pelvis the whole length of the limb, to which was attached a foot piece to keep the foot from rotating, and was used in addition to the plaster bandage.

I shall speak in a future article of the ingenious manner in which the plaster of Paris dressings are used in the hospitals in Paris.

W. S. Caldwell.

Paris, October 29, 1878.

---

High Temperature—Mr. Searle, of Scarborough, recently brought before the Clinical Society of London a case in which the temperature reached 50° C. (122° F.), and yet the patient recovered.

